# Supplementary material for: From sequence to enzyme mechanism using multi-label machine learning
Source: BMC Bioinformatics. 2014 May 19;15:150. doi: 10.1186/1471-2105-15-150 (PMC4229970; doi:10.1186/1471-2105-15-150)
Supplement: Additional file 2 — Java code of ml2db. Additional file ml2db_code.tar.gz contains the Java source code to run the multi-label machine learning experiments and save the results to database. The code’s Javadoc is included. [file 1471-2105-15-150-S2.zip › additional file 2/ml2db/ml2db/doc/main/ml2db/al/database/AlExperimentsTable.html]

AlExperimentsTable


JavaScript is disabled on your browser.


- Overview
- Package
- Class
- Use
- Tree
- Deprecated
- Index
- Help

- Prev Class
- Next Class

- Frames
- No Frames

- All Classes

- Summary:
- Nested |
- Field |
- Constr |
- Method

- Detail:
- Field |
- Constr |
- Method


main.ml2db.al.database

## Class AlExperimentsTable

- java.lang.Object
- - uk.ac.standrews.utils.main.database.Table
  - - main.ml2db.al.database.AlExperimentsTable

- ---

    

  ```
  public class AlExperimentsTable
  extends uk.ac.standrews.utils.main.database.Table
  ```

  Represents a table and table column names for storing details of an active
  learning experiment, such as: labelled and unlabelled arff file, algorithm
  used for evaluation, distance function used, utility function used etc.

  Version:
  :   12 Jul 2011

  Author:
  :   Luna De Ferrari ldf(at)st-andrews.ac.uk

- - ### Field Summary

    Fields

    | Modifier and Type | Field and Description |
    | --- | --- |
    | `static java.lang.String` | `ALGORITHM` the name for the algorithm table column |
    | `static java.lang.String` | `DISTANCE_FUNCTION` a string describing the distance function used to calculate the distance between two instances |
    | `static java.lang.String` | `END_TIMESTAMP` time at end of computation |
    | `static java.lang.String` | `EXPERIMENTS_TABLE_NAME` the name for the table to store the evaluation results |
    | `static java.lang.String` | `LABELLED_ARFF_ID` the table column to store the arff file id containing the labelled instances |
    | `static java.lang.String` | `START_TIMESTAMP` time at beginning of computation |
    | `static java.lang.String` | `UNLABELLED_ARFF_ID` the table column to store the arff file id containing the unlabelled instances (in active learning, labelled instances to be "treated" as unlabelled) |
    | `static java.lang.String` | `UTILITY_FUNCTION` a string describing the utility metric used to pick the next instance to be labelled |

    - ### Fields inherited from class uk.ac.standrews.utils.main.database.Table

      `m_writer`
  - ### Constructor Summary

    Constructors

    Constructor and Description

    `AlExperimentsTable(uk.ac.standrews.utils.main.database.DbManager manager)`
  - ### Method Summary

    Methods

    | Modifier and Type | Method and Description |
    | --- | --- |
    | `uk.ac.standrews.utils.main.database.TableRow` | `createRow(int labelledArffId, int unlabelledArffId, java.lang.String startTime, MulanLearner learner, java.lang.String distanceFunction, java.lang.String utilityFunction, uk.ac.standrews.utils.main.database.TableManager tableManager)` Create a row with the experiment details |
    | `uk.ac.standrews.utils.main.database.TableRow` | `createRow(java.lang.Object[] values, uk.ac.standrews.utils.main.database.TableManager tableManager)` |
    | `static java.util.Vector<uk.ac.standrews.utils.main.database.TableColumn>` | `getColumnDefinitions()` |

    - ### Methods inherited from class uk.ac.standrews.utils.main.database.Table

      `getColumnNames, getColumns, getDbManager, getTableCreator, getTableName, getTableReader, getTableRow, getTableWriter, hasKey, initialise, initialise, name, saveRow`
    - ### Methods inherited from class java.lang.Object

      `equals, getClass, hashCode, notify, notifyAll, toString, wait, wait, wait`

- - ### Field Detail


    - #### ALGORITHM

      ```
      public static final java.lang.String ALGORITHM
      ```

      the name for the algorithm table column

      See Also:
      :   Constant Field Values


    - #### LABELLED\_ARFF\_ID

      ```
      public static final java.lang.String LABELLED_ARFF_ID
      ```

      the table column to store the arff file id containing the labelled
      instances

      See Also:
      :   Constant Field Values


    - #### UNLABELLED\_ARFF\_ID

      ```
      public static final java.lang.String UNLABELLED_ARFF_ID
      ```

      the table column to store the arff file id containing the unlabelled
      instances (in active learning, labelled instances to be "treated" as
      unlabelled)

      See Also:
      :   Constant Field Values


    - #### END\_TIMESTAMP

      ```
      public static final java.lang.String END_TIMESTAMP
      ```

      time at end of computation

      See Also:
      :   Constant Field Values


    - #### DISTANCE\_FUNCTION

      ```
      public static final java.lang.String DISTANCE_FUNCTION
      ```

      a string describing the distance function used to calculate the distance
      between two instances

      See Also:
      :   Constant Field Values


    - #### UTILITY\_FUNCTION

      ```
      public static final java.lang.String UTILITY_FUNCTION
      ```

      a string describing the utility metric used to pick the next instance to
      be labelled

      See Also:
      :   Constant Field Values


    - #### EXPERIMENTS\_TABLE\_NAME

      ```
      public static final java.lang.String EXPERIMENTS_TABLE_NAME
      ```

      the name for the table to store the evaluation results

      See Also:
      :   Constant Field Values


    - #### START\_TIMESTAMP

      ```
      public static final java.lang.String START_TIMESTAMP
      ```

      time at beginning of computation

      See Also:
      :   Constant Field Values
  - ### Constructor Detail


    - #### AlExperimentsTable

      ```
      public AlExperimentsTable(uk.ac.standrews.utils.main.database.DbManager manager)
      ```
  - ### Method Detail


    - #### createRow

      ```
      public uk.ac.standrews.utils.main.database.TableRow createRow(int labelledArffId,
                                                           int unlabelledArffId,
                                                           java.lang.String startTime,
                                                           MulanLearner learner,
                                                           java.lang.String distanceFunction,
                                                           java.lang.String utilityFunction,
                                                           uk.ac.standrews.utils.main.database.TableManager tableManager)
      ```

      Create a row with the experiment details

      Parameters:
      :   `labelledArffId` -
      :   `unlabelledArffId` -
      :   `startTime` -
      :   `endTime` -
      :   `learner` -
      :   `distanceFunction` -
      :   `utilityFunction` -
      :   `tableManager` -

      Returns:


    - #### createRow

      ```
      public uk.ac.standrews.utils.main.database.TableRow createRow(java.lang.Object[] values,
                                                           uk.ac.standrews.utils.main.database.TableManager tableManager)
      ```


    - #### getColumnDefinitions

      ```
      public static java.util.Vector<uk.ac.standrews.utils.main.database.TableColumn> getColumnDefinitions()
      ```


- Overview
- Package
- Class
- Use
- Tree
- Deprecated
- Index
- Help

- Prev Class
- Next Class

- Frames
- No Frames

- All Classes

- Summary:
- Nested |
- Field |
- Constr |
- Method

- Detail:
- Field |
- Constr |
- Method
